# Metagenomic insights to bacterial communities, functional traits, and soil health in banana smallholder agroecosystems of Kenya

**DOI:** 10.3389/fmicb.2025.1582271

**Published:** 2025-05-30

**Authors:** Eugene Mwanza Muzami, George Kitundu, Oscar Mwaura Muriithi, Agnes Mumo Kavoo, Virginia Gathoni Gichuru, Wilton Mwema Mbinda

**Affiliations:** ^1^Department of Biochemistry and Biotechnology, Pwani University, Kilifi, Kenya; ^2^Department of Biological Sciences, Pwani University, Kilifi, Kenya; ^3^Department of Horticulture, Jomo Kenyatta University of Agriculture and Technology, Juja, Kenya; ^4^Pwani University Biosciences Research Centre (PUBReC), Pwani University, Kilifi, Kenya

**Keywords:** bacteria, banana rhizosphere, metagenomics, soil physicochemical properties, sustainable agriculture

## Abstract

Microbes inhabiting the banana rhizosphere are varied and mediate essential functions that enhance plant growth and increase crop productivity. Their abundance in soil habitats is a potential indicator of soil health and quality. Despite the well-known benefits of rhizosphere microorganisms in banana cultivation, their genomic and functional diversity remains largely unexplored within smallholder agroecosystems. In this study, we characterized the community composition and functional potential of bacteria in banana rhizospheric soils from Gituamba, Mangu and Ngenda constituencies in Kiambu County, Kenya. Using Illumina Novaseq sequencing, we analyzed 16S rRNA gene amplicons and shotgun metagenomic profiles to explore these microbial communities. Variations of soil physicochemical parameters across the study sites were assessed. The parameters varied across the sites, with soils in Gituamba and Ngenda depicting better soil fertility characteristics than Mangu. Amplicon sequencing profiles revealed higher bacterial diversity in Gituamba compared to Mangu, while the single sample from Ngenda exhibited moderate diversity. The dominant phyla across the study sites were *Proteobacteria, Actinobacteria*, and *Cyanobacteria*. Functional profiling of 16S rRNA gene amplicons showed a higher enrichment in Gituamba compared to Mangu. Overall, the functional profiling indicated that predicted metabolic pathways across the study sites were linked to genes encoded by the members of the most abundant bacterial phyla in the soil environments, majorly contributing to beneficial roles for soil health and crop yield. This study offers methods to reveal the banana rhizosphere as a rich reservoir for potential microbes of agricultural and biotechnological significance, which can promote sustainable agriculture.

## Introduction

Banana (*Musa paradisiaca*) is a key crop in the global food industry, ranking among the top fruit crops in terms of both production and economic value across the world (Alemu, 2017). It is a vital staple food with high nutritional benefits attributed to its richness in potassium, carbohydrates and vitamin A (Muthee et al., 2019). Bananas serve as a staple food for both rural and urban populations in Kenya (Wahome et al., 2021). Banana cultivation is an important economic activity in Kenya. However, small-scale farmers' production does not meet the current market demand (Wahome et al., 2023). The low production is due to numerous challenges such as agronomic practices, poor soil quality and the impact of pests and diseases (Rossmann et al., 2012). Numerous studies suggest that factors impacting soil biodiversity, including changes in land use, depletion of soil organic matter, and land degradation, have contributed to the decline of both aboveground and belowground microbiome abundance (Dixit et al., 2024). These factors directly affect banana production and have led to increased attention to assess soil health, particularly bacterial composition in rhizosphere soil habitats. This reflects the understanding that soil bacteria are essential for improving the overall soil and plant production (Giri and Varma, 2020). A diverse array of microbes is crucial for soil functions, as these communities significantly contribute to sustainable agricultural practices. Therefore, soil microorganisms are recognized as mediators of numerous processes that enhance agricultural productivity.

Plants and their related microorganisms interact to create communities of genotypes known as holobionts (Sarkar et al., 2022). These microbes influence the health and functioning of the plant by modifying the supply of essential nutrients and resisting both biotic and abiotic stresses (Alawiye and Babalola, 2021). Plant roots not only grant mechanical support, but also facilitate absorption of water and nutrients, and exudate various chemical substances that enhance plant-microbiome interactions (Molefe et al., 2021). The rhizosphere is the narrow region that surrounds the roots of plants and houses numerous microorganisms that define variety of anabolic and catabolic activities within soils. Plants release organic compounds into the rhizosphere through root exudates, shaping complex metabolic interactions among organisms. These interactions can support plant growth, remain neutral, or even be detrimental, influencing the surrounding microbial community (Omotayo et al., 2021).

Soil hosts a large community of microbes per gram of soil which include fungi, bacteria, viruses and protozoa in the rhizosphere. Among rhizobiomes, bacteria are the most abundant and serve as key markers of soil health and productivity concerning their swift responses to alternating environmental changes (Enagbonma et al., 2020). Rhizospheric bacterial communities promote growth through various mechanisms, both directly and indirectly. Rhizosphere bacteria promote nitrogen fixation, serve as biological control agents against pathogens in the soil, synthesize phytohormones and generate compounds like siderophores, antibiotics, cyanides, and ammonia (Alawiye and Babalola, 2019). The distribution of soil bacteria in various agroecosystems is primarily influenced by soil parameters such as soil texture, organic matter, organic carbon, soil nutrients and pH. Given that rhizospheric bacteria are highly responsive to alterations in the soil environment, shifts in their community structure indicate variations in soil biological activity and overall soil quality (He et al., 2023).

Although numerous studies have explored the banana root microbiome (Birt et al., 2022; Kaushal et al., 2020a,b), the relationship between soil microorganisms and soil health, particularly within smallholder banana farming systems, remains poorly understood. Identifying key microorganisms that shape the rhizosphere and understanding their functional traits are essential for advancing knowledge of soil-plant interactions in these agroecosystems. Moreover, shifts in the abundance and functional capabilities of soil bacterial communities under different farming conditions are still not well-characterized. To address these gaps, meta-omic approaches utilizing next-generation sequencing technologies are increasingly applied to analyze environmental metagenomes, allowing for the identification of microbial taxa and the discovery of functionally important genes and bioactive compounds (Nwachukwu and Babalola, 2022). In this study, we employed Illumina NovaSeq high throughput 16S rRNA gene amplicon and shotgun metagenomic sequencing to characterize the taxonomic composition and functional potential of bacterial communities in rhizosphere soils from smallholder banana farms in Kiambu County, Kenya. We also assessed variations in soil physicochemical properties across the study sites. We hypothesized that differences in soil physicochemical properties among sites would be associated with distinct bacterial community structures and functional profiles in the banana rhizosphere.

## Materials and methods

### Study site

**Figure 1:**
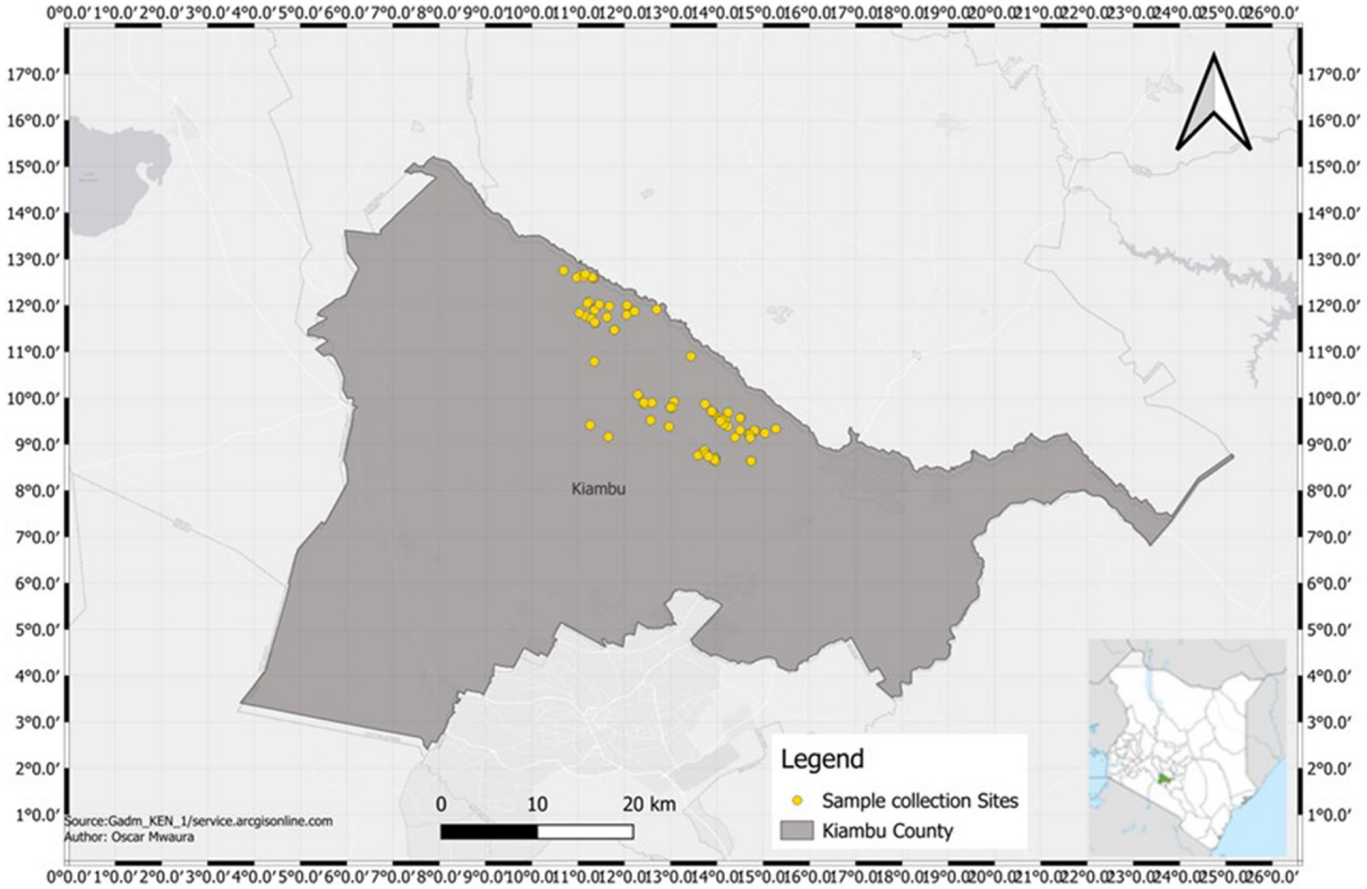
Map of soil sampling sites within Gatundu North and Gatundu South sub-counties in Kiambu County, Kenya.

The study was conducted in Kiambu County (36°49′0.0″E and 1°10′0.0″S) located in Kenya's central region (Figure 1) and covers a total area of 2,543.5 km^2^ (Kutwa et al., 2016). It has four broad topographical zones: upper highland, lower highland, upper midland and lower midland zones (Njiru, 2019). Three study sites were investigated within the lower highland zone: Gituamba and Mangu wards in Gatundu North sub-County and Ngenda ward in Gatundu South sub-County. The County is characterized by an annual average rainfall of 1,200 mm and an annual mean temperature of 26°C with temperatures ranging from 7°C in the upper highland areas to 34°C in the lower midland zone. The altitude of the County lies between 1,500 and 1,800 m above sea level and is generally a tea and dairy zone with agricultural activities like maize, horticultural crops and sheep farming. The average relative humidity ranges from 54% in the dry months and 300% in the wet months of March up to August. A bi-modal type of rainfall: short rains (mid-October to November) and long rains (mid-March to May) are experienced in the County. The major categories of soils in the County are high-level upland soils, plateau soils and volcanic footbridge soils. These soils are of varying fertility levels with soils from high-level uplands, which are from volcanic rocks, being very fertile. Coffee and tea are the main cash crops in the county while the main food crops grown in the county include maize, beans, pineapples, potatoes, vegetables and bananas. In Kiambu County, banana production is primarily practiced in high-rainfall areas that have been under continuous cultivation for extended periods, often resulting in decreased yields (Nzioka, 2009).

### Collection of soil samples

Sampling was conducted according to the described method by Wahome et al. (2023) in October 2022. Sampling occurred in smallholder banana farms, where most farmers practice mixed cropping systems, typically integrating bananas with other crops such as maize, beans, and vegetables. Across all farms, banana plants sampled were mature, characterized by fruiting bunches, ensuring consistency in the plant growth stage and minimizing variability due to plant age or vegetative state. Rhizospheric soil samples were collected at a depth of 15–20 cm at ~100 m spacing from each collection point using a sterile hand shovel applying the cluster sampling method. Soil sampling was carried out across and diagonally from 26 points per sampling site in each study site. A total of 78 soil samples of 200 g each were mixed and packed in sterile Ziplock bags in a dry iced box before they were transported and stored at −20°C at the Pwani University Biosciences Research Center (PUBReC) lab for further analysis. One portion (half) of each of the 78 soil samples was air-dried at room temperature for 1 week and sieved through a 100-mesh sifter to remove stones and visible plant fragments. The soils were stored at 4°C for further physicochemical analysis at the soil mechanics laboratory at Jomo Kenyatta University of Agriculture and Technology. The remaining halves of the soil samples were pooled into three composite samples representing each study site and stored at −80°C for soil genomic DNA extraction at Pwani University Biosciences Research Center (PUBReC).

### Soil physicochemical analysis

The measured soil parameters mainly included: the soil pH, soil water electrical conductivity, total organic carbon, organic matter, nitrogen, potassium and phosphorus content. Soil pH was measured using a soil pH meter (FE28, Mettler Toledo, Zurich, Switzerland) with a soil–water ratio of 1:2.5 (w/v) (Han et al., 2023). The electrical conductivity (EC) of the soil was measured using a portable analyzer (YSI, USA) (Tan et al., 2023). Total organic carbon (TOC) and available nitrogen (N) were determined by the sulphuric acid/potassium dichromate (H_2_SO_4_-K_2_Cr_2_O_7_) wet oxidation and the Kjeldahl methods, respectively (Li et al., 2022). TOC was analyzed using Shimadzu TOC-L CPH while N was measured using Lachat QuikChem 8500 equipment. Available phosphorus (P) was analyzed by inductively coupled ICP-OES Optima 8000 plasma-optical emission spectroscopy (Perkin Elmer, Massachusetts, USA) (Li et al., 2022). The soil organic matter (OM) was measured using the potassium dichromate external heating method (Tan et al., 2023) while available potassium (K) was extracted using the flame photometric method via digestion with ammonium acetate (NH_4_OAC) using the Cole-Parmer (USA) Flame Photometer 1265 (Han et al., 2023).

### DNA extraction and sequencing

Total soil DNA was extracted by the cetyltrimethylammonium bromide (CTAB) method. The purity and quality of the genomic DNA samples were checked using 1.2% agarose gel electrophoresis, and the concentrations were measured using a NanoDrop 2000 spectrophotometer (Thermo Fisher, Massachusetts, USA) before shipping the samples to Macrogen, South Korea for 16S rRNA gene amplicon and shotgun metagenomic sequencing using the Illumina Novaseq 6000 sequencing platform. The first two DNA samples (Gituamba and Mangu) were subjected to amplicon sequencing where the hypervariable region (V3–V4) was amplified using the 16S amplicon rRNA primer pair 515F (5′-GTGYCAGCMGCCGCGGTAA-3′) and 806R (5′-GGACTACNVGGGTWTCTAAT-3′) while the third DNA sample (Ngenda) was subjected to shotgun metagenomic sequencing. For amplicon sequencing, libraries were created, and each sample underwent a single-step PCR with 35 cycles using the HotStarTaq Plus Master Mix Kit (Qiagen, Valencia, CA). The PCR conditions were as follows: an initial denaturation at 95°C for 10 min, then 35 cycles comprising denaturation at 95°C for 30 s, annealing at 53°C for 40 s, and extension at 72°C for 1 min, with a final extension at 72°C for 10 min. After PCR, all amplicons from the samples were purified using SPRI beads. For shotgun metagenomic sequencing, library preparation followed the protocol for the Nextera DNA Flex kit (Illumina). Briefly, the sample began with 50 ng of DNA, which was fragmented and then tagged with Illumina sequencing adapters. Amplification of the libraries involved six PCR cycles. After amplification, library concentration was determined with the Qubit^®^ dsDNA HS Assay Kit (Life Technologies), and average fragment size was assessed using the Agilent 2100 Bioanalyzer (Agilent Technologies). The libraries were then pooled at a final concentration of 0.7 nM in equimolar amounts and sequenced in a paired-end format for 300 cycles on the NovaSeq 6000 platform (Illumina). The paired-end raw reads for all three samples were stored in FASTQ format.

### Sequence analysis, taxonomic and functional classification

#### Taxonomic classification of 16S rRNA profiles

Quantitative Insights into Microbial Ecology (QIIME v2024.2) was used to process the raw amplicon sequences (Bolyen et al., 2019). Raw data quality control was done using FASTQC v0.11.9 (Chen et al., 2017). Raw sequence data was imported into QIIME as a manifest file using the q2-tools import plugin followed by quality filtering that involved adapter removal from the paired-end demultiplexed sequences using the q2-Cutadapt trim-paired plugin. Subsequently, the reads were denoised using the Divisive Amplicon Denoising Algorithm (DADA2). The q2-dada2 denoise-paired plugin was used to filter the paired-end reads based on the quality score and length of the sequences as well as the removal of chimeras and dereplicated sequences. This was followed by the calculation of denoising statistics that produced files containing a summary of denoising results, representative sequences, and the feature count table. Further, the q2-feature classifier and assigned taxonomy plugin were used to classify the representative sequences. First, a machine learning approach involving a Scikit-learn multinomial naïve Bayes classifier was trained on an integrated reference database: Greengenes, SILVA and RDP database (GSR-DB) (Molano et al., 2024). The classifier was trained on our target hypervariable region of interest, the V3–V4 of 16S rRNA sequences. Secondly, the classified representative sequences were assigned taxonomy using the trained classifier. Taxonomy barplots were created using the q2-taxa barplot plugin. A *de novo* phylogeny-based approach was used to create a phylogenetic tree using the MAFFT-FastTree method in which a multiple sequence alignment with MAFFT was followed by building the maximum-likelihood rooted tree with FastTree using the q2-phylogeny plugin. Alpha diversity metrics (Observed features, Shannon index, and phylogenetic distance) were calculated after samples were subsampled without replacement (rarefied) to 225,541 sequences per sample using the q2-diversity alpha-rarefaction plugin. Data was presented as a direct visualization of QIIME2 artifacts on QIIME2 View website (https://view.qiime2.org).

#### Functional prediction of 16S rRNA profiles

Predictive functional profiling of bacterial communities was conducted using Phylogenetic Investigation of Communities by Reconstruction of Unobserved States2 (PICRUSt2) v2.5.2 (Douglas et al., 2020). Representative sequences were aligned using HMMER followed by placement into a reference tree utilizing EPA-NG (Barbera et al., 2018) and gappa (Czech et al., 2020). The number of 16S rRNA gene copies was normalized and gene families were inferred with Castor, a tool for hidden state prediction (Ye and Doak, 2009). These predicted gene families were subsequently mapped to MetaCyc pathways using MinPath (Ye and Doak, 2009).

#### Taxonomic and functional classification of shotgun metagenomic sequencing profiles

Shotgun sequencing data was analyzed with the SqueezeMeta program v1.6.3, a fully automated pipeline for metagenomics that covered all steps of the analysis (Tamames and Puente-Sánchez, 2019). Adapter removal, quality filtering and trimming of the reads were done by Trimmomatic (Bolger et al., 2014). The high-quality reads were assembled into contigs using MEGAHIT v1.2.9 (Li et al., 2015) via the sequential mode. Prinseq was used to remove short contigs (<200 bp) and determine the contig statistics (Schmieder and Edwards, 2011). The contigs were subjected to gene prediction using Prodigal software v2.6.3, which was employed to retrieve the corresponding amino acid sequences (Hyatt et al., 2010). Diamond v2.1.10 (Buchfink et al., 2021) was used to search for similarity between the NCBI nr database (Sayers et al., 2019). Taxonomic assignments of the genes were implemented using the lowest common ancestor (LCA) algorithm of the hits for each query gene searched against the reference database, NCBI nr. For functional assignment, Diamond was also used to compare gene sequences against the KEGG Orthology database (Kanehisa et al., 2017).

### Statistical analyses

All statistical analyses were conducted in R v4.3.3 (Kelley et al., 2008). A one-way analysis of variance (ANOVA) was used to evaluate the differences in soil physicochemical properties across the study sites and Tukey's pairwise comparison was used for the means at a significant level (*p*-value < 0.05). Spearman's correlation coefficient method was employed to assess the correlation between soil pH and other soil physicochemical properties. The diversity within the bacterial communities in the amplicon sequence data was depicted qualitatively using observed features, Shannon and phylogenetic distance (PD) matrices which were visualized as alpha rarefaction plots. Vegan package in R (Dixon, 2003) was used to evaluate the alpha diversity of the bacterial community in the shotgun metagenomic sequence data using the Shannon and Simpson indexes. The sequencing quality monitoring (SQM) tools package in R (Puente-Sánchez et al., 2020) was used to analyze both taxonomic and functional profiling data generated from the SqueezeMeta pipeline. Default parameters were used for all software unless otherwise specified.

## Results

### Soil physicochemical properties analysis

**Figure 2:**
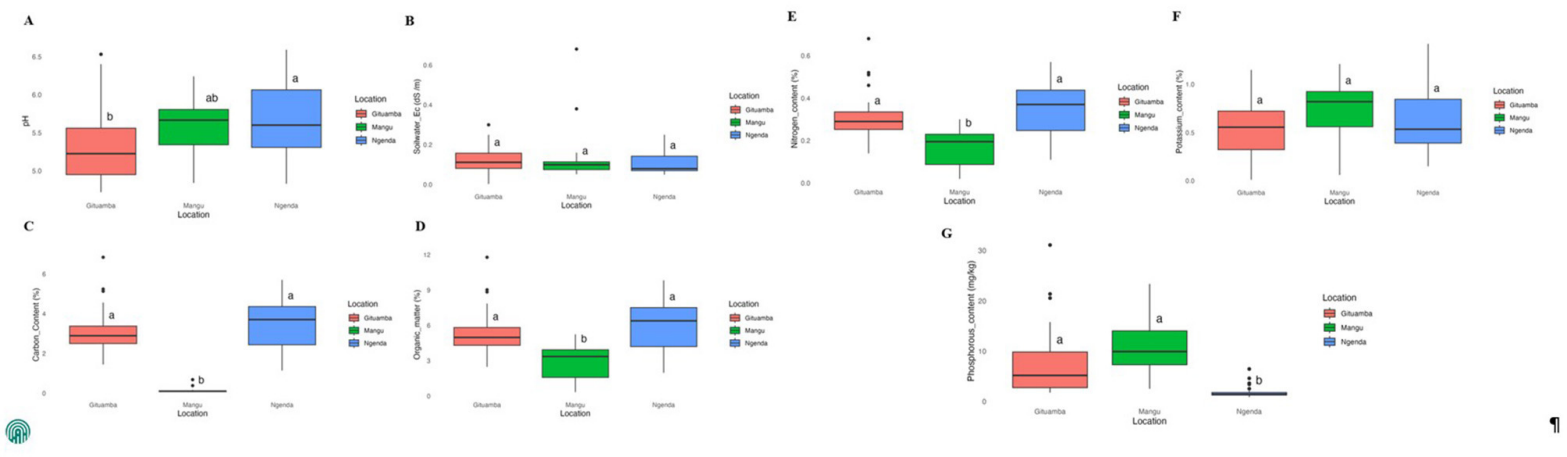
Box plots showing the means of soil physicochemical properties for Gituamba, Mangu and Ngenda: **(A)** pH, acidity/basicity; **(B)** EC, soil water Electrical conductivity; **(C)** %TOC, percentage of total organic carbon; **(D)** %O.M, percentage of organic matter; **(E)** %N, percentage of available nitrogen; **(F)** %K, percentage of available potassium; **(G)** P_2_O_5_ (mg/kg), available phosphorus content. Different superscript letters indicate significant differences between treatments (*p* < 0.05) according to the Tukey honest significance test (HSD). Outliers represent data points that deviated significantly from each individual the sample dataset.

Results of the means of soil physicochemical properties of the composite samples revealed that the soil pH was slightly acidic across the sites. Soils in Gituamba had the lowest pH (5.32 ± 0.47) while Ngenda recorded the highest pH (5.67 ± 0.51; Supplementary material 1). There was no significant difference in pH between Mangu and other study sites (Figure 2A). Mangu soils contained a slightly higher EC value (0.13 ± 0.13 *dS*/*m*) while Ngenda had the lowest value (0.11 ± 0.01 *dS*/*m*). The differences in EC across the three sites were not significant (Figure 2B). The contents of TOC were highest in Ngenda soils (3.49 ± 1.30%), while Mangu soils exhibited the lowest content (1.77 ± 0.87%; Figure 2C). Similarly, Ngenda soils had the highest content of OM (6.02 ± 2.24%) while Mangu soils had a significantly lower content (3.05 ± 1.50%) as compared to Gituamba and Ngenda respectively (Figure 2D). The highest content of available N was contained in Ngenda soils (0.35 ± 0.13%) while Mangu had a significantly lower available N (0.18 ± 0.09%) compared to Gituamba and Ngenda respectively (Figure 2E). The levels of available K content were relatively similar and insignificant across all the sites with the highest content recorded in Mangu soils (0.64 ± 0.33%; Figure 2F). A higher amount of available P content was contained in Mangu (10.56 ± 5.69 mg/kg) while the lowest content was recorded in Ngenda soils (1.89 ± 1.29 mg/kg; Figure 2G).

### Spearman's correlation analysis

**Figure 3:**
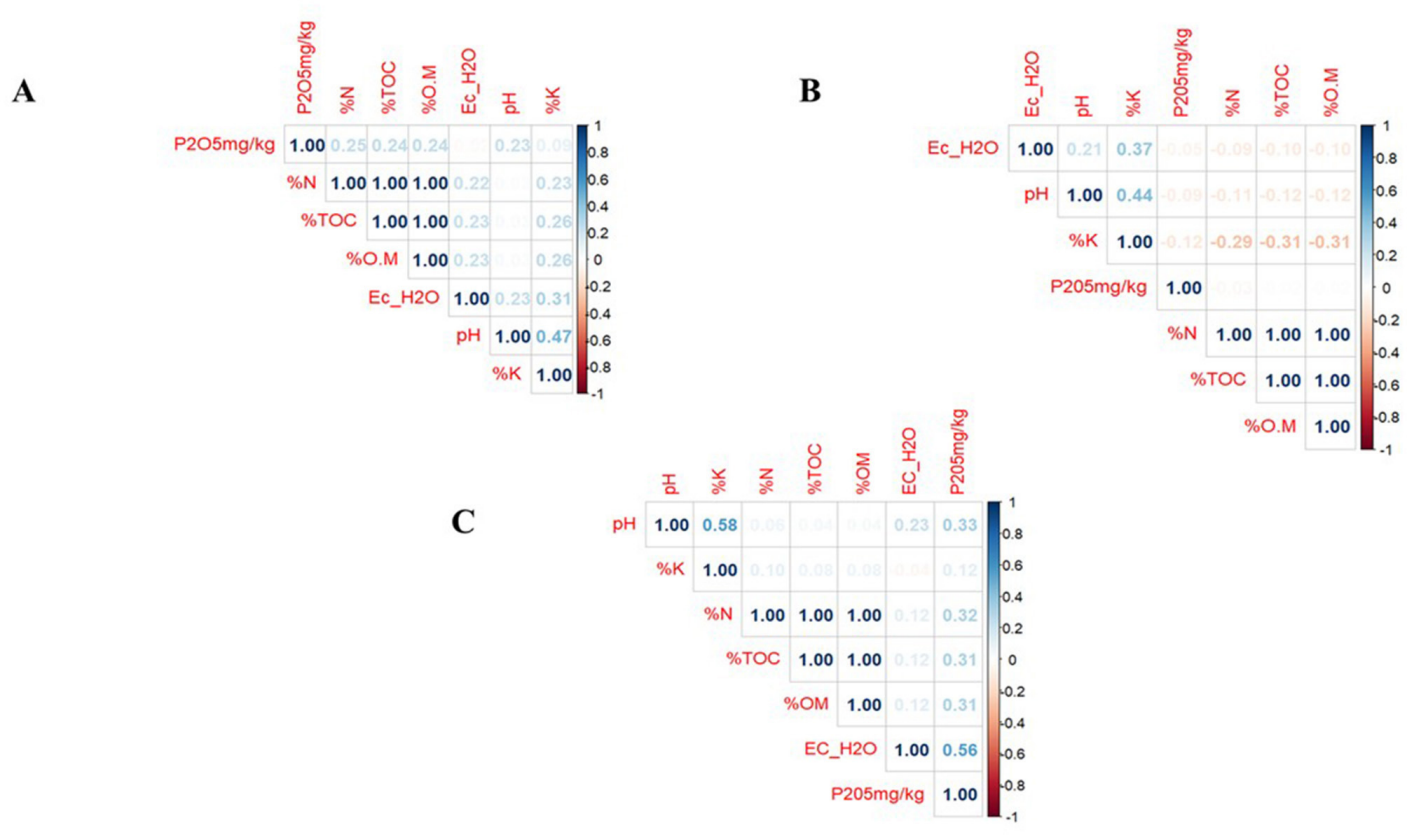
Spearman's correlation of the relationship between soil pH and other soil physicochemical properties (*p* < 0.05) in **(A)** Gituamba, **(B)** Mangu, and **(C)** Ngenda.

The relationship between soil pH and most of the soil parameters [EC, %TOC, %O.M, %N and P (mg/kg)] in Gituamba soils exhibited weak positive correlations indicating that changes in pH did not greatly affect other parameters. Available potassium levels showed a moderate correlation to soil pH suggesting that an increase in pH resulted in a noticeable increase in potassium content in Gituamba (Figure 3A). In Mangu soils, there was a weak negative correlation between pH and other soil parameters [%TOC, %O.M, %N, and P (mg/kg)] while a weak positive correlation was recorded between pH and EC. Like Gituamba soils, pH positively correlated with available potassium content (Figure 3B). The soil pH in Ngenda soils had weak positive correlations with other soil parameters (EC, %TOC, %O.M, and %N) as well as moderate positive correlations with available potassium and phosphorus were observed (Figure 3C).

### Analysis of sequencing data

A total of 2,769,898 16S rRNA sequence reads were obtained from the two samples. Sample 1 (Gituamba) had 1,887,650 reads while sample 2 (Mangu) had the least number 882,248 reads. The percentage of reads that passed quality filtering for the two samples was 90.32 and 89.09%, respectively (Supplementary material 2). A total of 67,136,628 shotgun metagenomic sequence reads were obtained from sample 3 (Ngenda) of which 27,584,941 (41.09%) reads were mapped and used for taxonomic and functional annotation. After assembly, 2,504,874 contigs were identified. The longest contig was 125,240 bp, while the shortest contig was 128 bp. A total of 3,205,501 open reading frames (ORFs) were predicted from Prodigal of which 1,508,761 were annotated against the KEGG Orthology database (Supplementary material 3).

### Bacterial diversity analysis

**Figure 4:**
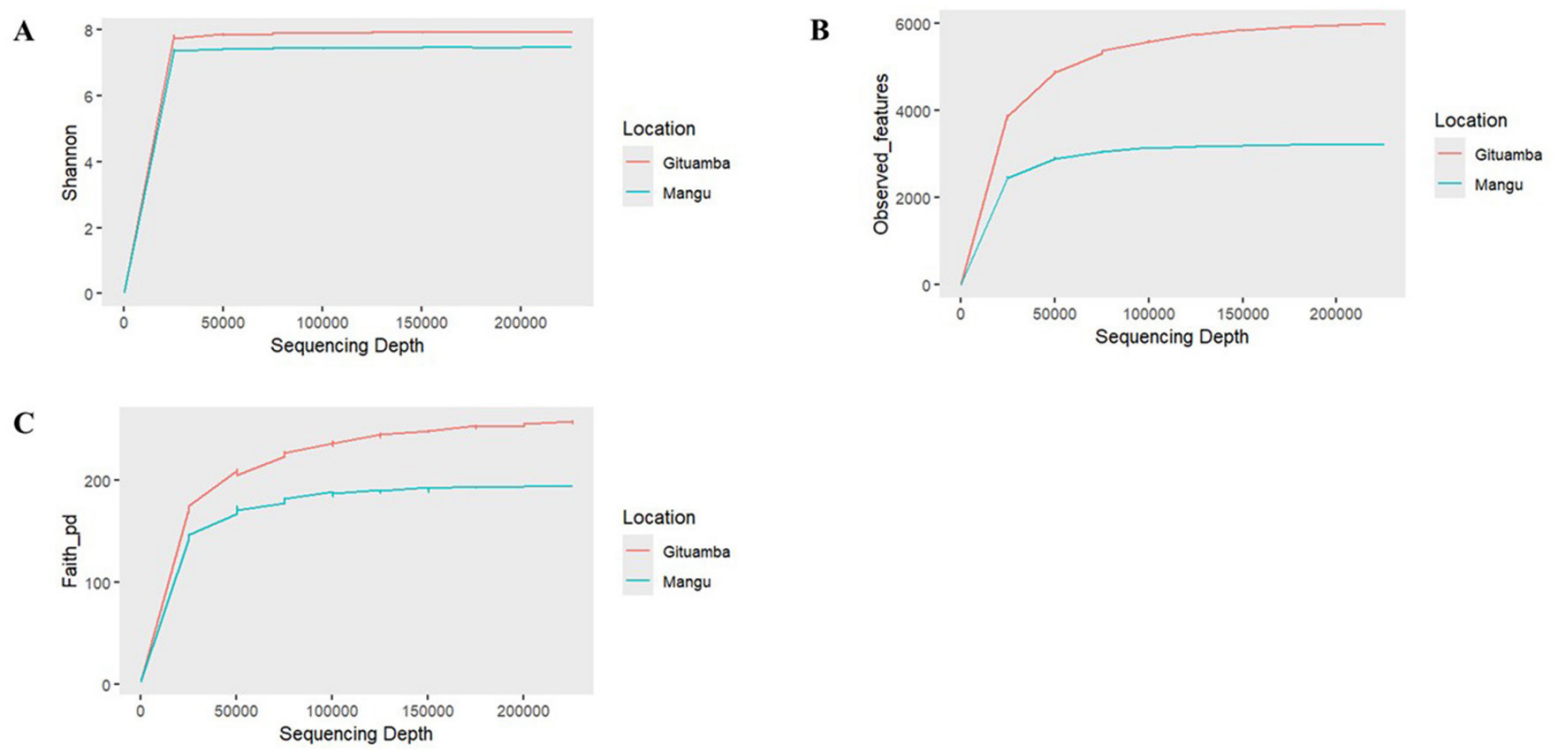
Alpha rarefaction plots showing bacterial diversity metrices as a function of sampling depth: **(A)** Shannon, **(B)** Observed_features, **(C)** Faith_pd in Gituamba and Mangu.

Based on Shannon diversity metrices, a higher bacterial diversity was recorded in Gituamba than Mangu (Figure 4A). The rarefaction plot of observed features revealed that Gituamba exhibited a higher number of observed features compared to Mangu, indicating a richer species composition (Figure 4B). The rarefaction plot based on phylogenetic distance (PD) produced similar results to Shannon and observed features, indicating greater phylogenetic distances among bacterial communities in Gituamba than Mangu (Figure 4C). Due to the limited number of amplicon samples (*n* = 1 per site), statistical comparison using *t*-tests was not appropriate. The lack of replication prevented reliable estimation of within-group variability. As a result, we focused on qualitative and descriptive analyses to highlight differences between sites. In the sample analyzed from Ngenda, the Shannon index (1.77) and Simpson index (0.49) (Supplementary material 4) indicated moderate microbial diversity. Nonetheless, given the lack of sample replication, these results are limited to the individual sample and should not be generalized to characterize the overall bacterial community at the site.

### Bacterial community composition

#### Bacterial community composition based on 16S rRNA profiles

**Figure 5:**
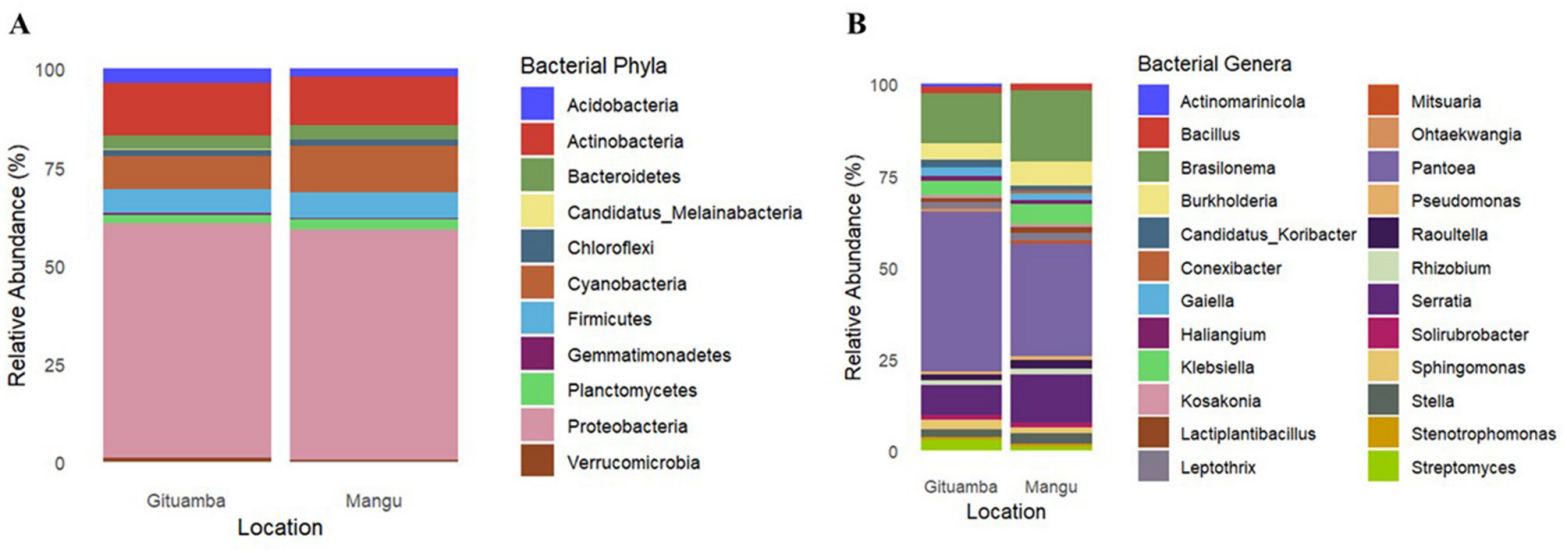
Relative abundance of bacterial community composition at different taxonomic levels: **(A)** Phylum and **(B)** Genus in Gituamba, and Mangu, respectively.

Classified representative amplicon sequence variants (ASVs) were assigned to 50 bacterial phyla, 117 classes, 247 orders, 508 families, 1,440 genera, and 2,748 species (Supplementary material 5). There were 11 dominant bacterial phyla in Gituamba and Mangu soils respectively. The most dominant phylum was *Proteobacteria* (59.7 and 58%) followed by *Actinobacteria* (13.5 and 12.4%), and *Cyanobacteria* (8.2 and 11.9%; Figure 5A). At the genus level, 24 prominent genera were observed in both samples. The most dominant were *Pantoea* (43.7 and 30.8%) and *Brasilonema* (13.6 and 19.4%; Figure 5B).

#### Bacterial community composition based on shotgun metagenomic sequencing profiles

**Figure 6:**
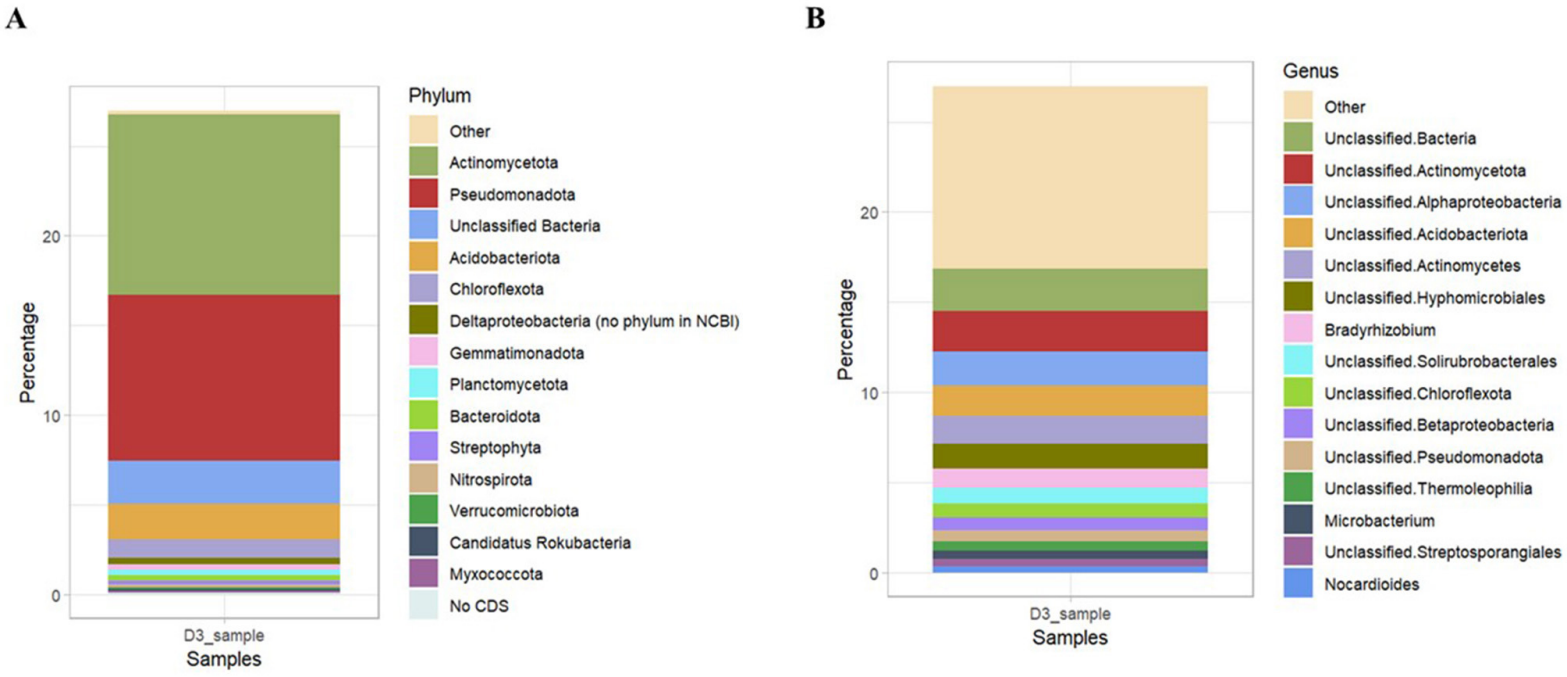
Relative abundance of bacterial community composition at different taxonomic levels: **(A)** Phylum and **(B)** Genus in Ngenda.

Assembly-based metagenome taxonomic profiling revealed that Ngenda soils had four superkingdoms: Bacteria, Archaea, Eukaryota, and Viruses. Bacteria was the most abundant taxa containing 98% of the total soil taxa. The bacterial community was composed of 102 distinct phyla, 238 classes, 435 orders, 808 families, 1,772 genera, and 2,791 species (Supplementary material 6). At the phylum level, *Actinobacteria* (37%) was the most abundant followed by *Pseudomonadota* (34%), *Acidobacteria* (7%), and *Chloroflexota* (3%; Figure 6A). At the genus level, *Bradyrhizobium* (4.0%) was the most abundant followed by *Actinomycetes* (2.0%; Figure 6B).

### Metagenomic functional prediction

#### Metagenomic functional prediction based on 16S rRNA profiles

**Figure 7:**
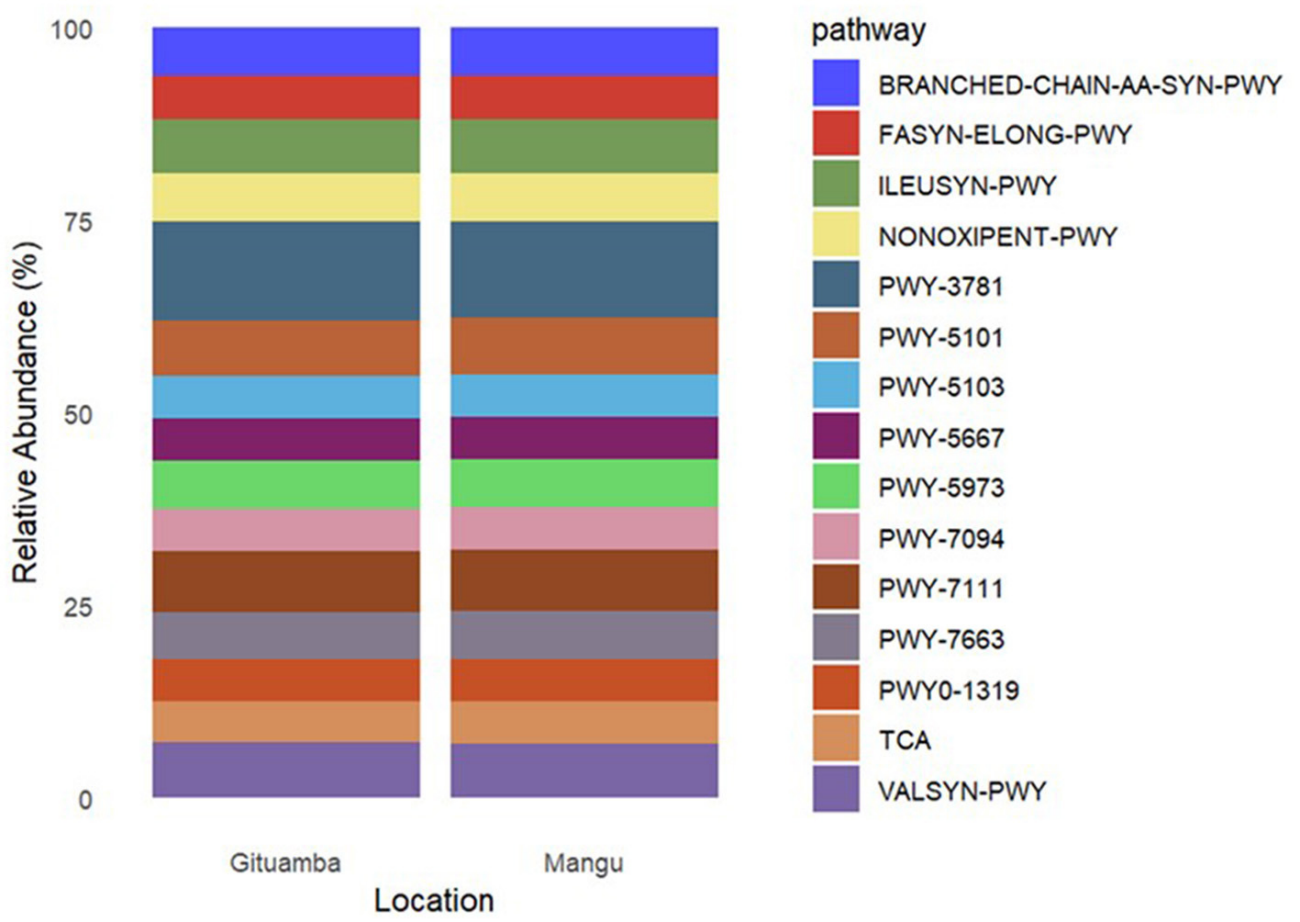
Relative abundance of the most abundant predicted MetaCyc pathways within the bacterial metagenome in Gituamba and Mangu.

A total of 7,634 KEGG orthologs (enzymes) predicted by PICRUSt2 were consolidated into 435 Meta-Cyc pathways. There were 15 most abundant functional pathways in both Gituamba and Mangu. PWY-3781 [Aerobic respiration I (cytochrome *c*)] was the most abundant followed by PWY-7111 (pyruvate fermentation to isobutanol), PWY-5101 (L-Isoleucine biosynthesis II), ILEUSYN-PWY (L-Isoleucine biosynthesis I from threonine) and VALSYN-PWY (L-Valine biosynthesis; Figure 7). Analysis of pathway differentiation by location demonstrated that the mean abundance of predicted pathways was significantly elevated in Gituamba relative to Mangu.

#### Metagenomic functional prediction based on shotgun metagenomic sequencing profiles

**Figure 8:**
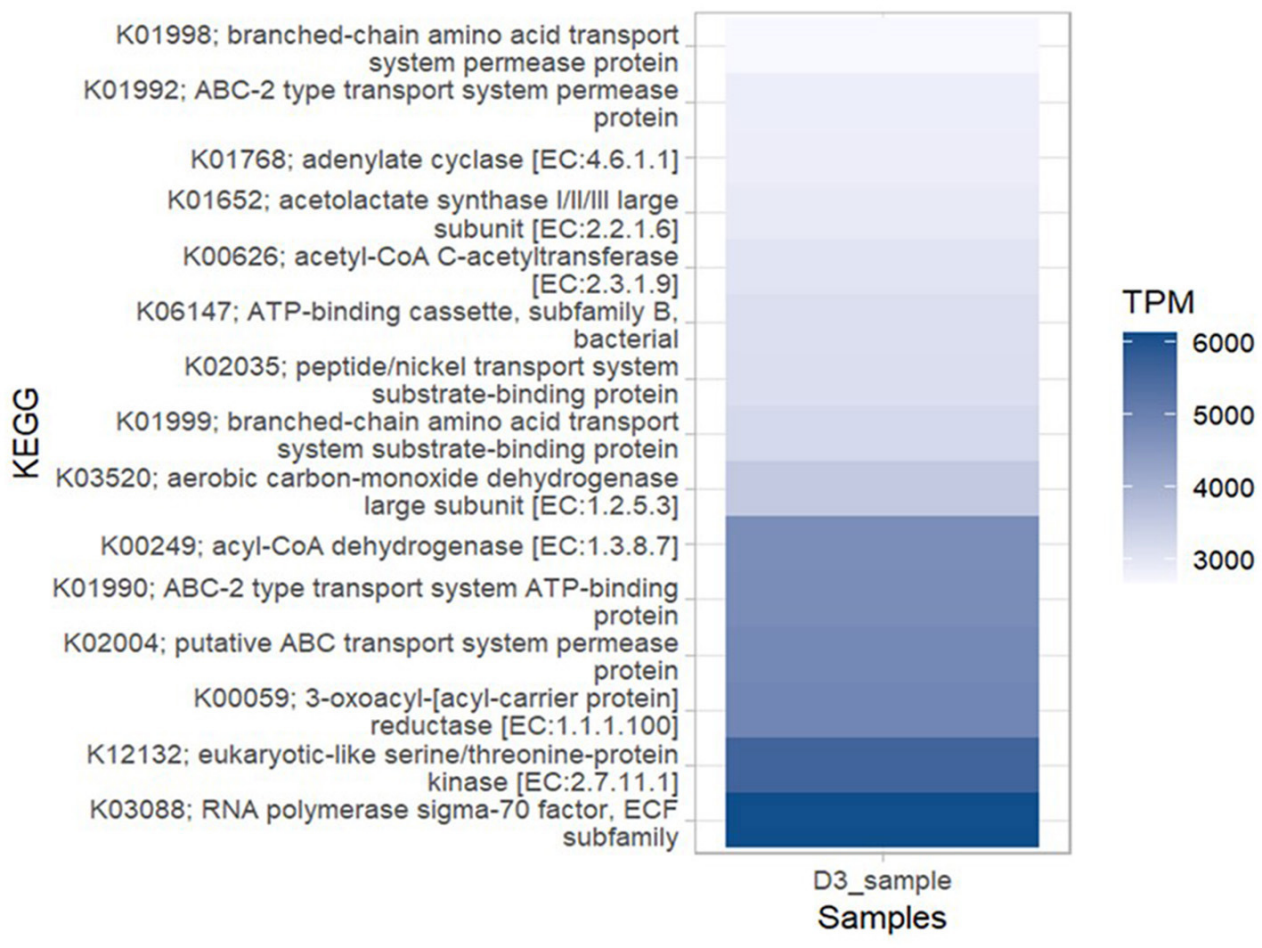
Most abundant KEGG orthologs of bacteria. Abundance of each function is counted in transcripts per million (TPM), genes from each function per million genes in the bacterial community metagenome in Ngenda.

**Figure 9:**
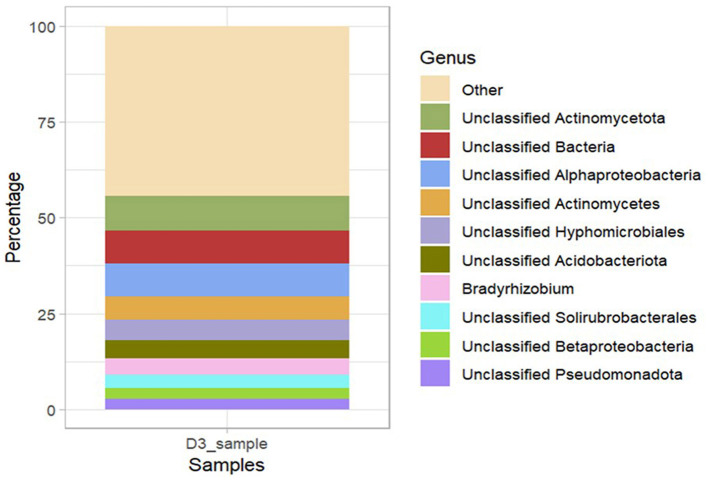
Taxonomy of bacteria at the genus level, showing the distribution of abundant bacteria containing the open reading frames (ORFs) that encode for the main biological metabolic pathways (primary level functions) in Ngenda soils.

A total of 9,585 KEGG orthologs (KOs)/Enzymes were predicted among which 15 of them were the most abundant KOs [higher number of TPM (transcripts per million)] within the bacterial community metagenome. The most abundant ortholog in the metagenome was: RNA polymerase sigma-70 factor, ECF subfamily (KEGG ID: K03088). Other abundant KOs included: eukaryotic-like serine/threonine-protein-kinase [EC:2.7.11.1] (KEGG ID: K12132) followed by 3-oxoacyl- [acyl-carrier protein] reductase [EC:1.1.1.100] (KEGG ID: K00059), putative ABC transport system permease protein (KEGG ID: K02004), ABC- 2 type transport system ATP- binding protein (KEGG ID:K01990), acyl-COA dehydrogenase [EC:1.3.8.7] (KEGG ID: K00249) and aerobic carbon-monoxide dehydrogenase large subunit [EC:1.2.5.3] (KEGG ID: K03520; Figure 8). The KOs predicted within the metagenome were associated with six high-level functions: metabolism, genetic information processing, cellular processes, environmental information processing, organismal systems and human diseases (Supplementary material 7). The key lower-level functions included amino acid, lipid, sulfur, energy, nucleotide, vitamins and carbohydrate metabolism. For genetic information processing, the prominent low-level functions were replication, translation, transcription, folding, sorting, and degradation. Cellular processes within the metagenome included cell growth and death, cell motility, cellular community and transport and catabolism while the key functions predicted under environmental information processing were signal transduction and membrane transport. The most abundant bacterial genera with open reading frames (ORFs) containing annotation of the six high-level functions were *Bradyrhizobium, Alphaproteobacteria, Betaproteobacteria, Pseudomonadota, Solirubacterales, Acidobacteria*, and *Hyphomicrobiales* (Figure 9).

## Discussion

Rhizosphere-inhabiting microbes can enhance plant growth and health (Kong and Liu, 2022). Their diversity in soil habitats is a potential indicator of soil health and quality (Bargali, 2024). However, changes in the diversity and functional potential of soil microbes within banana farming systems are still not well-understood. We used Illumina Novaseq, a high-throughput sequencing approach, to examine the bacterial communities in the rhizosphere of banana plants grown in small-scale farms in Kiambu, focusing on their composition and functional potential. Also, the variation of the soil physicochemical parameters within our study sites was assessed.

Physicochemical analysis revealed that soil pH was slightly acidic across all study sites, with non-significant differences (*p*-value > 0.05) between Mangu and the other locations. The observed pH values in Mangu and Ngenda fall within the optimal range of 5.5–7.5 for banana cultivation, as reported by Nyamamba et al. (2020). Soil pH plays a crucial role in regulating chemical reactions and nutrient availability in the soil, directly impacting plant growth (Cordero et al., 2020). In addition, it is the primary factor that determines the microbial community structure in natural environmental systems (Kang et al., 2013). Our results align with the research conducted by Swafo and Dlamini (2022), who observed slightly acidic soils for banana production upon characterizing the soil parameters. The variations in soil nutrient properties (TOC, N, and OM) showed a similar trend across our study sites. Previous reports suggest that the higher levels of soil nutrients in Gituamba and Ngenda can be attributed to high biomass production by the banana plants and other crops, mulching practices, effective crop residue management and favorable soil microbial activities (Gerke, 2022; Githongo et al., 2022; Mushtaq et al., 2023). Spearman's correlation revealed that soil pH had weak correlations with most of the parameters (EC, TOC, OM, N, and P) in our study sites suggesting that soil pH did not strongly affect most soil parameters. Contrary to our findings, a related study by Zhang et al. (2019) revealed that soil pH showed positive correlations with other soil parameters in different soil horizons, making it a key factor in controlling soil nutrient availability. The findings of our study suggest that soil pH management practices should be prioritized across our study sites to enhance sustainable banana production. Overall, soils in Gituamba and Ngenda depicted better soil fertility characteristics with higher TOC, OM, and N levels compared to Mangu. The variations in soil parameters across the study sites could be due to variations in soil management practices, farming techniques, soil types, climate, and soil microbial communities (Singh et al., 2020).

Since rhizospheric soil is closest to the roots, it provides an avenue to explore microbial communities (Sui et al., 2019). From our metagenomics analyses of rhizospheric bacterial communities and their potential functions, we hypothesized that diverse microbial populations in banana soils play beneficial roles in supporting plant health. Metagenomic profiling, as demonstrated in this study, facilitates the identification of previously unknown bacterial taxa and their functional roles. These bacteria are essential for boosting plant growth, strengthening disease resistance, and enhancing soil health. Additionally, we anticipate that these microbial communities will display distinct biogeographical patterns across different banana-growing regions, influenced by variations in soil characteristics, climate, and agricultural practices. The makeup of microbial communities in rhizospheric soil is strongly linked to soil quality and the overall health of crops (Shi et al., 2021). Soil-associated microbiota significantly influences how plants adapt and respond to environmental changes, including stressors like salinity and drought (Zhang et al., 2017).

In Gituamba and Mangu agroecosystems, our primary goal was to characterize the bacterial composition and diversity. This guided the choice of amplicon sequencing, particularly 16S rRNA gene sequencing, which provided a cost-effective and high-throughput method to profile the microbial communities at the taxonomic level. On the other hand, the Ngenda agroecosystems soil was subjected to shotgun metagenomic sequencing. Due to relatively better soil fertility characteristics in Ngenda compared to other sites, we aimed to go beyond taxonomic classification to analyze the functional traits and metabolic potential of the microbial communities. Shotgun metagenomics thus allowed whole genome reconstruction and functional annotation, providing detailed insights into the potential roles of soil bacteria in ecosystem attributes. By using both amplicon and shotgun sequencing approaches, we ensured a balanced approach in characterizing the taxonomic composition in all sites while obtaining more functional insights in Ngenda than Gituamba and Mangu.

Phylum *Proteobacteria* was the most prevalent across all our sites and has been consistently observed in studies evaluating bacterial communities in the banana rhizosphere (Fu et al., 2017; Kaushal et al., 2022a,b; Mia et al., 2010). Members of the phylum *Proteobacteria*, a Gram-negative bacterium, are also enriched in the rhizosphere soil of many plants (Fu et al., 2022; Gao et al., 2019; Zhang et al., 2022). They play an active role in organic matter decomposition, ammonia oxidation, nitrogen fixation and degradation of inorganic compounds (Ling et al., 2022). The phylum *Actinobacteria*, a group of Gram-positive bacteria, plays a crucial role in decomposing organic matter and produces antibiotics that inhibit plant pathogens in soil (Dhakal et al., 2017). In addition, *Cyanobacteria, Planctomycetes*, and *Chloroflexi* are known to play a role in nitrogen cycling within agroecosystems (Li et al., 2021). A previous study by Beltran-Garcia et al. (2021) also identified key processes mediated by *Proteobacteria, Actinobacteria*, and *Cyanobacteria* within banana agroecosystems.

The predominant genus, *Pantoea*, a Gram-negative bacterium belongs to the *Enterobacteriaceae* family, and its members are versatile biocontrol agents, protecting against pathogenic microbes in several plant varieties (Duchateau et al., 2024). Other abundant genera including *Brasilonema*, a genus of *Cyanobacteria*, play a significant role in banana soils, particularly in mitigating Panama disease, a soil-borne infection, caused by pathogenic fungi, *Fusarium*. Other genera including *Serratia, Klebsiella, Burkholderia*, and *Streptomyces* contribute to plant growth promotion by production of phytohormones, mitigation of plant abiotic stresses and inhibition of plant pathogens (Dos Santos et al., 2022; Kulkova et al., 2024; Olanrewaju and Babalola, 2019). The bacterial communities in Ngenda soils exhibited a moderate level of alpha diversity, suggesting that the bacterial community diversity was moderate with a balanced representation of different species. This suggests a relatively healthy and stable ecosystem. Phyla *Pseudomonadota* and *Actinobacteria* were the most prominent in Ngenda soils, consistent with findings by Liu et al. (2022) who investigated the vertical distribution patterns of soil microbial communities in peatlands under varying environmental conditions. Members of *Actinobacteria* bacteria are an important phylum that act as plant growth promoters due to their vital role in the cycling of organic matter and inhibition of several plant pathogens (Ibrahimi et al., 2023). At the genus level, we found *Bradyrhizobium* as the most abundant in Ngenda soils and its members actively promote plant growth and yield by fixing nitrogen in soil (Shahrajabian et al., 2021). A study by Chalasani et al. (2021) also reported the positive roles of *Bradyrhizobium* enhancing legume nodulation, which helps to maintain plant community structure and restore degraded ecosystems. A previous study by Wahome et al. (2023) explored the abundance of soil bacteria as well the effects of soil parameters on their distribution in small-holder banana farms in Kisii, Nyamira, and Embu Counties of Kenya. Similar to our findings, they reported *Proteobacteria* phylum as the most prevalent. They also noted a variation in soil pH which affects the bacterial community composition in all their study sites. Overall, their study revealed the importance of soil health and management practices in shaping microbial diversity, which in turn affects the productivity of banana agroecosystems in these regions. In our study, the adaptability of soil bacteria likely accounts for their significant presence in the study sites and underscores their potential role in maintaining soil health and enhancing crop yield. These sensitive bacterial species may be crucial for evaluating the impact of human activities on banana microbiomes and their associated ecological functions.

The functional diversity of soil microbes is a vital indicator of community composition and ecological roles, essential for understanding the contributions of microorganisms in different environments (Chen et al., 2020). We focused on bacterial functional predictions of pathways and enzymes encoded within bacterial metagenomes. Metabolic gene functions in the 16S rRNA gene sequences revealed a significantly higher abundance in Gituamba than in Mangu. Metabolic pathways such as aerobic respiration I (PWY-3781), pyruvate fermentation to isobutanol (PWY-711), L-isoleucine biosynthesis II (PWY-5101), L-isoleucine biosynthesis I from threonine (ILEUSYN-PWY), and L-valine biosynthesis (VALSYN-PWY) were prominent. These findings resonate with studies by Tang et al. (2024) and Zhou et al. (2023) who reported that most bacterial metabolic pathways in soils were associated with carbon IV oxide assimilation, acetate, carbohydrates, amino acids biosynthesis, and inorganic compounds degradation. In our study, most of the predicted pathways are linked to crucial processes such as nitrate reduction and the breakdown of various anaerobic aromatic compounds, including catechol, creatinine, toluene, salicylate, L-arabinose, L-valine, and the biosynthesis of adenosine nucleotides, 1,5-anhydrofructose, and pyrimidine ribonucleotides (Zhou et al., 2023). Therefore, the predicted pathways, based on PICRUSt2 analysis, are associated with functions that may contribute to plant growth, development, and the synthesis of bioactive substances. However, it is important to note that these predictions are based on 16S rRNA gene profiles and do not represent direct functional measurements. Previous studies have linked similar microbial functions, such as energy metabolism, fermentation/alternative energy metabolism and amino acid biosynthesis pathways, to enhanced plant performance (Adedayo et al., 2022). Recent reports indicate that the most common bacterial phyla that contain metabolic pathways in rhizospheric soils that contribute to soil health and plant growth include *Proteobacteria, Actinobacteria, Cyanobacteria, Acidobacteria, Bacteroidetes, Firmicutes, Chloroflexi, and Planctomycetes* (Li et al., 2014, 2020; Lopes et al., 2016; Nicolas et al., 2021).

The most abundant KEGG Orthologs/Enzymes within the bacterial metagenome in Ngenda were RNA polymerase sigma-70 factor, Extracytoplasmic function (ECF) subfamily, eukaryotic-like serine/threonine-protein-kinase, 3-oxoacyl- [acyl-carrier protein] reductase, putative ABC transport system permease protein, ATP-binding cassette (ABC)-2 type transport system ATP-binding protein, acyl-CoA dehydrogenase and aerobic carbon-monoxide dehydrogenase large subunit. These results align with a similar investigation by Wang et al. (2023) that explored the microbial communities of rhizosphere soil and root endophytes in *Panax notoginseng* from various geographical regions. The KEGG pathways in this study site are associated with key processes that contribute to improved soil health and crop production in agroecosystems as demonstrated by Abulfaraj et al. (2024). By connecting taxonomy and function, we discovered that genes possessed by *Bradyrhizobium, Actinomycetota, Alphaproteobacteria, Betaproteobacteria, Pseudomonadota, Solirubacterales, Acidobacteria*, and *Hyphomicrobiales* were associated with metabolic pathways for metabolism, genetic information processing, cellular processes, environmental information processing, organismal systems and human disease processes. Analyzing the functional microbiome revealed potential bacteria that could benefit plants and promote their growth at our study locations.

While functional prediction provided initial insights into the contribution of microbial communities to gene functions in banana rhizospheric soils, it is recommended to apply several omics approaches such as metagenomics, metatranscriptomics, metabolomics and proteomics in future studies. These approaches can help address the known biases associated with functional prediction tools. Also, the selection between shotgun and amplicon sequencing methods for microbiome analysis typically depends on the specific research objectives. For example, amplicon sequencing is ideal for analyzing larger samples, such as in longitudinal studies, but it provides limited taxonomic and functional resolution (Jovel et al., 2016). In contrast, shotgun metagenomics generally offers more comprehensive information on numerous genes, resulting in enhanced taxonomic and functional resolution of sequences (Peterson et al., 2021; Zaheer et al., 2018). In the current study, both approaches revealed almost similar taxonomic groups whereas functional profiling yielded more information in the shotgun approach compared to 16S rRNA gene sequencing. The findings from our study describe the need for the application of omics techniques to provide more research insights in banana-soil microbiome interactions, for sustainable agriculture in different growing regions.

## Conclusion

This study provides baseline data on the bacterial composition and predicted gene functions associated with the rhizospheres of banana plants in Kenya. It also highlights differences in soil physicochemical properties across agroecosystems, emphasizing the need for future research to explore how these factors influence microbial community structure. Through amplicon and shotgun metagenomic sequencing, we demonstrated that the abundance of beneficial rhizosphere bacteria across diverse environments is linked to their ecological functions, contributing to soil health and crop productivity. A comprehensive understanding of key microbial metabolic pathways that facilitate plant development could empower farmers to adopt sustainable soil management practices, enhancing both environmental and economic outcomes.

However, we acknowledge certain limitations in this study, including the use of only two composite amplicon samples per site without biological replication and the application of shotgun metagenomics to a single sample. These constraints limit the generalizability and robustness of our diversity and functional predictions. Future research should incorporate multiple biological replicates and broader shotgun metagenomic analyses to strengthen statistical validity and provide a more comprehensive understanding of microbial roles in the banana rhizosphere. Expanding such efforts could reveal novel microbial taxa and functions with potential applications in ecosystem management, agriculture, and biotechnology.

## Data Availability

Sequences from this study were deposited into the SRA under the BioProject ID PRJNA1146306 (https://www.ncbi.nlm.nih.gov/search/all/?term=PRJNA1146306).

## References

[B1] Abulfaraj A. A. Shami A. Y. Alotaibi N. M. Alomran M. M. Aloufi A. S. Al-Andal A.. (2024). Exploration of genes encoding KEGG pathway enzymes in rhizospheric microbiome of the wild plant *Abutilon fruticosum*. AMB Express 14:27. 10.1186/s13568-024-01678-4 38381255 PMC10881953

[B2] Adedayo A. Fadiji A. Babalola O. (2022). The effects of plant health status on the community structure and metabolic pathways of rhizosphere microbial communities associated with *Solanum lycopersicum*. Horticulturae 8:404. 10.3390/horticulturae8050404

[B3] Alawiye T. Babalola O. (2019). Bacterial diversity and community structure in typical plant rhizosphere. Diversity 11:179. 10.3390/d11100179

[B4] Alawiye T. Babalola O. (2021). Metagenomic insight into the community structure and functional genes in the sunflower rhizosphere microbiome. Agriculture 11:167. 10.3390/agriculture11020167

[B5] Alemu M. M. (2017). Banana as a cash crop and its food security and socioeconomic contribution: the case of Southern Ethiopia, Arba Minch. J. Environ. Prot. 08, 319–329. 10.4236/jep.2017.83024

[B6] Barbera P. Kozlov A. M. Czech L. Morel B. Darriba D. Flouri T.. (2018). Data from: EPA-ng: massively parallel evolutionary placement of genetic sequences (Version 1, p. 16601287 bytes) [Dataset]. Dryad. 10.1101/291658 PMC6368480 30165689

[B7] Bargali S. S. (2024). Soil microbial biomass: a crucial indicator of soil health. Curr. Agric. Res. J. 12, 01–06. 10.12944/CARJ.12.1.01

[B8] Beltran-Garcia M. J. Martinez-Rodriguez A. Olmos-Arriaga I. Valdez-Salas B. Chavez-Castrillon Y. Y. Di Mascio P.. (2021). Probiotic endophytes for more sustainable banana production. Microorganisms 9:1805. 10.3390/microorganisms9091805 34576701 PMC8469954

[B9] Birt H. W. G. Pattison A. B. Skarshewski A. Daniells J. Raghavendra A. Dennis P. G. (2022). The core bacterial microbiome of banana (*Musa* spp.). *Environ. Microbiome* 17:46. 10.1186/s40793-022-00442-0 36076285 PMC9461194

[B10] Bolger A. M. Lohse M. Usadel B. (2014). Trimmomatic: a flexible trimmer for Illumina sequence data. Bioinformatics 30, 2114–2120. 10.1093/bioinformatics/btu170 24695404 PMC4103590

[B11] Bolyen E. Rideout J. R. Dillon M. R. Bokulich N. A. Abnet C. C. Al-Ghalith G. A.. (2019). Reproducible, interactive, scalable and extensible microbiome data science using QIIME 2. Nat. Biotechnol. 37, 852–857. 10.1038/s41587-019-0209-9 31341288 PMC7015180

[B12] Buchfink B. Reuter K. Drost H.-G. (2021). Sensitive protein alignments at tree-of-life scale using DIAMOND. Nat. Methods 18, 366–368. 10.1038/s41592-021-01101-x 33828273 PMC8026399

[B13] Chalasani D. Basu A. Pullabhotla S. V. Jorrin B. Neal A. L. Poole P. S.. (2021). Poor competitiveness of *Bradyrhizobium* in pigeon pea root colonization in Indian soils. MBio 12, 10–1128. 10.1128/mBio.00423-21 34225488 PMC8406239

[B14] Chen S. Huang T. Zhou Y. Han Y. Xu M. Gu J. (2017). AfterQC: automatic filtering, trimming, error removing and quality control for fastq data. BMC Bioinformatics 18(S3):80. 10.1186/s12859-017-1469-3 28361673 PMC5374548

[B15] Chen Z.-J. Shao Y. Li Y.-J. Lin L.-A. Chen Y. Tian W.. (2020). Rhizosphere bacterial community structure and predicted functional analysis in the water-level fluctuation zone of the danjiangkou reservoir in china during the dry period. Int. J. Environ. Res. Public Health 17:1266. 10.3390/ijerph17041266 32079120 PMC7068437

[B16] Cordero J. De Freitas J. R. Germida J. J. (2020). Bacterial microbiome associated with the rhizosphere and root interior of crops in Saskatchewan, Canada. Can. J. Microbiol. 66, 71–85. 10.1139/cjm-2019-0330 31658427

[B17] Czech L. Barbera P. Stamatakis A. (2020). Genesis and Gappa: processing, analyzing and visualizing phylogenetic (placement) data. Bioinformatics 36, 3263–3265. 10.1093/bioinformatics/btaa070 32016344 PMC7214027

[B18] Dhakal D. Pokhrel A. R. Shrestha B. Sohng J. K. (2017). Marine rare actinobacteria: isolation, characterization, and strategies for harnessing bioactive compounds. Front. Microbiol. 8:1106. 10.3389/fmicb.2017.01106 28663748 PMC5471306

[B19] Dixit M. Ghoshal D. Lal Meena A. Ghasal P. C. Rai A. K. Choudhary J.. (2024). Changes in soil microbial diversity under present land degradation scenario. Total Environ. Adv. 10:200104. 10.1016/j.teadva.2024.20010434042469

[B20] Dixon P. (2003). VEGAN, a package of R functions for community ecology. J. Veg. Sci. 14, 927–930. 10.1111/j.1654-1103.2003.tb02228.x

[B21] Dos Santos I. B. Pereira A. P. D. A. De Souza A. J. Cardoso E. J. B. N. Da Silva F. G. Oliveira J. T. C.. (2022). Selection and characterization of burkholderia spp. for their plant-growth promoting effects and influence on maize seed germination. Front. Soil Sci. 1:805094. 10.3389/fsoil.2021.805094

[B22] Douglas G. M. Maffei V. J. Zaneveld J. R. Yurgel S. N. Brown J. R. Taylor C. M.. (2020). PICRUSt2 for prediction of metagenome functions. Nat. Biotechnol. 38, 685–688. 10.1038/s41587-020-0548-6 32483366 PMC7365738

[B23] Duchateau S. Crouzet J. Dorey S. Aziz A. (2024). The plant-associated Pantoea spp. as biocontrol agents: mechanisms and diversity of bacteria-produced metabolites as a prospective tool for plant protection. Biol. Control 188:105441. 10.1016/j.biocontrol.2024.105441

[B24] Enagbonma B. J. Ajilogba C. F. Babalola O. O. (2020). Metagenomic profiling of bacterial diversity and community structure in termite mounds and surrounding soils. Arch. Microbiol. 202, 2697–2709. 10.1007/s00203-020-01994-w 32725600

[B25] Fu L. Penton C. R. Ruan Y. Shen Z. Xue C. Li R.. (2017). Inducing the rhizosphere microbiome by biofertilizer application to suppress banana *Fusarium wilt* disease. Soil Biol. Biochem. 104, 39–48. 10.1016/j.soilbio.2016.10.008

[B26] Fu L. Yan Y. Li X. Liu Y. Lu X. (2022). Rhizosphere soil microbial community and its response to different utilization patterns in the semi-arid alpine grassland of northern Tibet. Front. Microbiol. 13:931795. 10.3389/fmicb.2022.931795 35935214 PMC9354816

[B27] Gao X. Wu Z. Liu R. Wu J. Zeng Q. Qi Y. (2019). Rhizosphere bacterial community characteristics over different years of sugarcane ratooning in consecutive monoculture. Biomed Res. Int. 2019, 1–10. 10.1155/2019/6916189 31815142 PMC6878781

[B28] Gerke J. (2022). The central role of soil organic matter in soil fertility and carbon storage. Soil Syst. 6:33. 10.3390/soilsystems6020033

[B29] Giri B. Varma A. (Eds.). (2020). Soil Health, Vol. 59. New York, NY: Springer International Publishing. 10.1007/978-3-030-44364-1

[B30] Githongo M. Kiboi M. Muriuki A. Fliessbach A. Musafiri C. Ngetich F. K. (2022). Organic carbon content in fractions of soils managed for soil fertility improvement in sub-humid agroecosystems of Kenya. Sustainability 15:683. 10.3390/su15010683

[B31] Han C. Zhang Z. Gao Y. Wang W. Chen J. Wang Y. (2023). Microbiome reveals the effects of biogas fertilizer on soil microbial community structure and diversity in perennial apple orchards. Horticulturae 9:1023. 10.3390/horticulturae9091023

[B32] He Z. Yuan C. Chen P. Rong Z. Peng T. Farooq T. H.. (2023). Soil microbial community composition and diversity analysis under different land use patterns in Taojia River Basin. Forests 14:1004. 10.3390/f14051004

[B33] Hyatt D. Chen G.-L. LoCascio P. F. Land M. L. Larimer F. W. Hauser L. J. (2010). Prodigal: prokaryotic gene recognition and translation initiation site identification. BMC Bioinformatics 11:119. 10.1186/1471-2105-11-119 20211023 PMC2848648

[B34] Ibrahimi M. Loqman S. Jemo M. Hafidi M. Lemee L. Ouhdouch Y. (2023). The potential of facultative predatory *Actinomycetota* spp. and prospects in agricultural sustainability. Front. Microbiol. 13:1081815. 10.3389/fmicb.2022.1081815 36762097 PMC9905845

[B35] Jovel J. Patterson J. Wang W. Hotte N. O'Keefe S. Mitchel T.. (2016). Characterization of the gut microbiome using 16S or shotgun metagenomics. Front. Microbiol. 7:459. 10.3389/fmicb.2016.00459 27148170 PMC4837688

[B36] Kanehisa M. Furumichi M. Tanabe M. Sato Y. Morishima K. (2017). KEGG: new perspectives on genomes, pathways, diseases and drugs. Nucleic Acids Res. 45, D353–D361. 10.1093/nar/gkw1092 27899662 PMC5210567

[B37] Kang S. Van Nostrand J. D. Gough H. L. He Z. Hazen T. C. Stahl D. A.. (2013). Functional gene array–based analysis of microbial communities in heavy metals-contaminated lake sediments. FEMS Microbiol. Ecol. 86, 200–214. 10.1111/1574-6941.12152 23710534

[B38] Kaushal M. Kolombia Y. Alakonya A. E. Kuate A. F. Ortega-Beltran A. Amah D.. (2022a). Subterranean microbiome affiliations of plantain (*Musa* spp.) under diverse agroecologies of Western and Central Africa. Microb. Ecol. 84, 580–593. 10.1007/s00248-021-01873-x 34585290 PMC9436888

[B39] Kaushal M. Mahuku G. Swennen R. (2020a). Metagenomic insights of the root colonizing microbiome associated with symptomatic and non-symptomatic Bananas in fusarium wilt infected fields. Plants 9:263. 10.3390/plants9020263 32085593 PMC7076721

[B40] Kaushal M. Swennen R. Mahuku G. (2020b). Unlocking the microbiome communities of banana (*Musa* spp.) under disease stressed (*Fusarium wilt*) and non-stressed conditions. Microorganisms 8:443. 10.3390/microorganisms8030443 32245146 PMC7144012

[B41] Kaushal M. Tumuhairwe J. B. Kaingo J. Richard M. Nakamanya F. Taulya G.. (2022b). Compositional shifts in microbial diversity under traditional banana cropping systems of Sub-Saharan Africa. Biology 11:756. 10.3390/biology11050756 35625484 PMC9138362

[B42] Kelley K. Lai K. Wu P.-J. (2008). “Using R for data analysis: a best practice for research,” in Best Practices in Quantitative Methods, ed. J. Osborne (London: SAGE Publications, Inc), 535–572. 10.4135/9781412995627.d40

[B43] Kong Z. Liu H. (2022). Modification of rhizosphere microbial communities: a possible mechanism of plant growth promoting rhizobacteria enhancing plant growth and fitness. Front. Plant Sci. 13:920813. 10.3389/fpls.2022.920813 35720594 PMC9198353

[B44] Kulkova I. Wróbel B. Dobrzyński J. (2024). *Serratia* spp. as plant growth-promoting bacteria alleviating salinity, drought, and nutrient imbalance stresses. Front. Microbiol. 15:1342331. 10.3389/fmicb.2024.1342331 38562478 PMC10982427

[B45] Kutwa A. A. Muhingi W. N. Kokonya D. (2016). Smallholder Rural Youth Farming in Kiambu County, Kenya. J. Cult. Soc. Devel. 25, 60–71

[B46] Li D. Liu C.-M. Luo R. Sadakane K. Lam T.-W. (2015). MEGAHIT: an ultra-fast single-node solution for large and complex metagenomics assembly via succinct *de Bruijn* graph. Bioinformatics 31, 1674–1676. 10.1093/bioinformatics/btv033 25609793

[B47] Li J. Wen Y. Yang X. (2021). Understanding the responses of soil bacterial communities to long-term fertilization regimes using DNA and RNA sequencing. Agronomy 11:2425. 10.3390/agronomy1112242527670433

[B48] Li M. Zhang K. Yan Z. Liu L. Kang E. Kang X. (2022). Soil water content shapes microbial community along gradients of wetland degradation on the Tibetan Plateau. Front. Microbiol. 13:824267. 10.3389/fmicb.2022.824267 35185848 PMC8847787

[B49] Li X. Rui J. Xiong J. Li J. He Z. Zhou J.. (2014). Functional potential of soil microbial communities in the maize rhizosphere. PLoS ONE 9:e112609. 10.1371/journal.pone.0112609 25383887 PMC4226563

[B50] Li Y. Wang C. Wang T. Liu Y. Jia S. Gao Y.. (2020). Effects of different fertilizer treatments on rhizosphere soil microbiome composition and functions. Land 9:329. 10.3390/land9090329

[B51] Ling N. Wang T. Kuzyakov Y. (2022). Rhizosphere bacteriome structure and functions. Nat. Commun. 13:836. 10.1038/s41467-022-28448-9 35149704 PMC8837802

[B52] Liu L. Wang Z. Ma D. Zhang M. Fu L. (2022). Diversity and distribution characteristics of soil microbes across forest–peatland ecotones in the permafrost regions. Int. J. Environ. Res. Public Health 19:14782. 10.3390/ijerph192214782 36429502 PMC9690085

[B53] Lopes L. D. Pereira E Silva M. D. C. Andreote F. D. (2016). Bacterial abilities and adaptation toward the rhizosphere colonization. Front. Microbiol. 7:1341. 10.3389/fmicb.2016.01341: 27610108 PMC4997060

[B54] Mia M. B. Shamsuddin Z. H. Wahab Z. Marziah M. (2010). Rhizobacteria as bioenhancer and biofertilizer for growth and yield of banana (*Musa* spp. cv.‘Berangan'). Sci. Hortic. 126, 80–87. 10.1016/j.scienta.2010.06.005

[B55] Molano L.-A. G. Vega-Abellaneda S. Manichanh C. (2024). GSR-DB: A manually curated and optimized taxonomical database for 16S rRNA amplicon analysis. mSystems 9:e00950-23. 10.1128/msystems.00950-23 38189256 PMC10946287

[B56] Molefe R. R. Amoo A. E. Babalola O. O. (2021). Metagenomic insights into the bacterial community structure and functional potentials in the rhizosphere soil of maize plants. J. Plant Interact. 16, 258–269. 10.1080/17429145.2021.1936228

[B57] Mushtaq H. Ganai B. A. Jehangir A. (2023). Exploring soil bacterial diversity in different micro-vegetational habitats of Dachigam National Park in North-western Himalaya. Sci. Rep. 13:3090. 10.1038/s41598-023-30187-w 36813837 PMC9947166

[B58] Muthee A. I. Gichimu B. M. Nthakanio P. N. (2019). Analysis of banana production practices and constraints in Embu County, Kenya. Asian J. Agric. Rural Dev. 9, 123–132. 10.18488/journal.1005/2019.9.1/1005.1.123.132

[B59] Nicolas A. M. Jaffe A. L. Nuccio E. E. Taga M. E. Firestone M. K. Banfield J. F. (2021). Soil candidate phyla radiation bacteria encode components of aerobic metabolism and co-occur with nanoarchaea in the rare biosphere of rhizosphere grassland communities. mSystems 6:e01205-20. 10.1128/msystems.01205-20 34402646 PMC8407418

[B60] Njiru E. B. K. (2019). Urban expansion and loss of agricultural land: A GIS based study of Kiambu County. Int. J. Sci. Res. 8, 915–919.

[B61] Nwachukwu B. C. Babalola O. O. (2022). Metagenomics: a tool for exploring key microbiome with the potentials for improving sustainable agriculture. Front. Sustain. Food Syst. 6:886987. 10.3389/fsufs.2022.886987

[B62] Nyamamba K. A. Ouna T. O. Kamiri H. Pane E. (2020). Effects of land use change on banana production: a case study of Imenti South Sub-County of Meru County in Kenya. BIoEx J. 2, 640–652. 10.33258/bioex.v2i3.303

[B63] Nzioka S. M. (2009). Economic efficiency analysis of banana farmers in Kiambu East District of Kenya: Technical inefficiency and marketing efficiency. J. Dev. Sustain. Agric. 4, 118–127. 10.11178/jdsa.4.118

[B64] Olanrewaju O. S. Babalola O. O. (2019). Streptomyces: implications and interactions in plant growth promotion. Appl. Microbiol. Biotechnol. 103, 1179–1188. 10.1007/s00253-018-09577-y 30594952 PMC6394478

[B65] Omotayo O. Igiehon O. Babalola O. (2021). Metagenomic study of the community structure and functional potentials in maize rhizosphere microbiome: elucidation of mechanisms behind the improvement in plants under normal and stress conditions. Sustainability 13:8079. 10.3390/su13148079

[B66] Peterson D. Bonham K. S. Rowland S. Pattanayak C. W. Resonance Consortium Klepac-Ceraj V. (2021). Comparative analysis of 16S rRNA gene and metagenome sequencing in pediatric gut microbiomes. Front. Microbiol. 12:670336. 10.3389/fmicb.2021.670336 34335499 PMC8320171

[B67] Puente-Sánchez F. García-García N. Tamames J. (2020). SQMtools: automated processing and visual analysis of ‘omics data with R and anvi'o. BMC Bioinformatics 21:358. 10.1186/s12859-020-03703-2 32795263 PMC7430844

[B68] Rossmann B. Müller H. Smalla K. Mpiira S. Tumuhairwe J. B. Staver C.. (2012). Banana-associated microbial communities in uganda are highly diverse but dominated by Enterobacteriaceae. Appl. Environ. Microbiol. 78, 4933–4941. 10.1128/AEM.00772-12 22562988 PMC3416381

[B69] Sarkar S. Kumar R. Kumar A. Kumar U. Singh D. K. Mondal S.. (2022). “Role of soil microbes to assess soil health,” in Structure and Functions of Pedosphere, eds. B. Giri, R. Kapoor, Q.-S. Wu, and A. Varma (New York, NY: Springer Nature Singapore), 339–363. 10.1007/978-981-16-8770-9_14

[B70] Sayers E. W. Cavanaugh M. Clark K. Ostell J. Pruitt K. D. Karsch-Mizrachi I. (2019). GenBank. Nucleic Acids Res. 47(D1), D94–D99. 10.1093/nar/gkz956 30365038 PMC6323954

[B71] Schmieder R. Edwards R. (2011). Quality control and preprocessing of metagenomic datasets. Bioinformatics 27, 863–864. 10.1093/bioinformatics/btr026 21278185 PMC3051327

[B72] Shahrajabian M. H. Sun W. Cheng Q. (2021). The importance of Rhizobium, Agrobacterium, Bradyrhizobium, Herbaspirillum, Sinorhizobium in sustainable agricultural production. Not. Bot. Horti Agrobot. Cluj-Napoca 49:12183. 10.15835/nbha49312183

[B73] Shi G. Sun H. Calderón-Urrea A. Li M. Yang H. Wang W.. (2021). Bacterial communities as indicators of soil health under a continuous cropping system. Land Degrad. Dev. 32, 2393–2408. 10.1002/ldr.3919

[B74] Singh S. Sharma A. Khajuria K. Singh J. Vig A. P. (2020). Soil properties changes earthworm diversity indices in different agro-ecosystem. BMC Ecol. 20:27. 10.1186/s12898-020-00296-5 32375784 PMC7203807

[B75] Sui X. Zhang R. Frey B. Yang L. Li M.-H. Ni H. (2019). Land use change effects on diversity of soil bacterial, Acidobacterial and fungal communities in wetlands of the Sanjiang Plain, northeastern China. Sci. Rep. 9:18535. 10.1038/s41598-019-55063-4 31811224 PMC6898332

[B76] Swafo S. M. Dlamini P. E. (2022). Unlocking the land capability and soil suitability of makuleke farm for sustainable banana production. Sustainability 15:453. 10.3390/su15010453

[B77] Tamames J. Puente-Sánchez F. (2019). SqueezeMeta, a highly portable, fully automatic metagenomic analysis pipeline. Front. Microbiol. 9:3349. 10.3389/fmicb.2018.03349 30733714 PMC6353838

[B78] Tan S. Jiang L. Liu J. Zeng Z. Xiao Y. Wu X.. (2023). Rhizosphere microorganisms and soil physicochemical properties of restored wetland plant communities at cutting slash of Populus deltoides in Dongting Lake. Wetlands 43:48. 10.1007/s13157-023-01696-1

[B79] Tang Y. Zhou S. Xiao Y. Zhang T. Tao X. Shi K.. (2024). Exploring the microbial ecosystem of *Berchemia polyphylla va*r. leioclada: a comprehensive analysis of endophytes and rhizospheric soil microorganisms. Front. Microbiol. 15:1338956. 10.3389/fmicb.2024.1338956 38544861 PMC10965698

[B80] Wahome C. N. Maingi J. M. Ombori O. Kimiti J. M. Njeru E. M. (2021). Banana production trends, cultivar diversity, and tissue culture technologies uptake in Kenya. Int. J. Agron 2021, 1–11. 10.1155/2021/6634046

[B81] Wahome C. N. Maingi J. M. Ombori O. Njeru E. M. Muthini M. Kimiti J. M. (2023). Diversity and abundance of bacterial and fungal communities in rhizospheric soil from smallholder banana producing agroecosystems in Kenya. Front. Hortic. 2:1061456. 10.3389/fhort.2023.1061456

[B82] Wang X. Jarmusch S. A. Frisvad J. C. Larsen T. O. (2023). Current status of secondary metabolite pathways linked to their related biosynthetic gene clusters in *Aspergillus* section Nigri. Nat. Prod. Rep. 40, 237–274. 10.1039/D1NP00074H 35587705

[B83] Ye Y. Doak T. G. (2009). A parsimony approach to biological pathway reconstruction/inference for genomes and metagenomes. PLoS Comput. Biol. 5:e1000465. 10.1371/journal.pcbi.1000465 19680427 PMC2714467

[B84] Zaheer R. Noyes N. Ortega Polo R. Cook S. R. Marinier E. Van Domselaar G.. (2018). Impact of sequencing depth on the characterization of the microbiome and resistome. Sci. Rep. 8:5890. 10.1038/s41598-018-24280-8 29651035 PMC5897366

[B85] Zhang H. Ullah F. Ahmad R. Ali Shah S. U. Khan A. Adnan M. (2022). Response of soil proteobacteria to biochar amendment in sustainable agriculture- a mini review. J. Soil Plant Environ. 1, 16–30. 10.56946/jspae.v1i2.56

[B86] Zhang H. Wang R. Chen S. Qi G. He Z. Zhao X. (2017). Microbial taxa and functional genes shift in degraded soil with bacterial wilt. Sci. Rep. 7:39911. 10.1038/srep39911 28051173 PMC5209727

[B87] Zhang Y.-Y. Wu W. Liu H. (2019). Factors affecting variations of soil pH in different horizons in hilly regions. PLoS ONE 14:e0218563. 10.1371/journal.pone.0218563 31216328 PMC6584057

[B88] Zhou L. Wu S. Ma M. (2023). First insights into diversity and potential metabolic pathways of bacterial and fungal communities in the rhizosphere of *Argemone mexicana* L. (Papaveraceae) from the water-level-fluctuation zone of Wudongde Reservoir of the upper Yangtze river, China. Biodivers. Data J. 11:e101950. 10.3897/BDJ.11.e101950 38327346 PMC10848652

