# Supplementary material for: Metagenomic insights to bacterial communities, functional traits, and soil health in banana smallholder agroecosystems of Kenya
Source: Front Microbiol. 2025 May 30;16:1582271. doi: 10.3389/fmicb.2025.1582271 (PMC12162901; doi:10.3389/fmicb.2025.1582271)

**Supplementary material**

**Supplementary material 1****: Means and standard deviations of soil physicochemical properties for Gituamba, Mangu and Ngenda**

|  | **Gituamba** | **Mangu** | **Ngenda** | **Pr(>F)** |
| --- | --- | --- | --- | --- |
| **pH** | 5.32 ± 0.47^a^ | 5.61 ± 0.38^ab^ | 5.67 ± 0.51^b^ | 0.021 |
| **EC (*dS* /*m*)** | 0.13 ± 0.07^a^ | 0.13 ± 0.13^a^ | 0.11 ± 0.01^a^ | 0.597 |
| **%TOC** | 3.13 ± 1.22^a^ | 1.77 ± 0.87^b^ | 3.49 ± 1.30^a^ | 2×10^-16^ |
| **%O.M** | 5.39 ± 2.10^a^ | 3.05 ± 1.50^b^ | 6.02 ± 2.24^a^ | 33×10^-6^ |
| **%N** | 0.31 ± 0.12^a^ | 0.18 ± 0.09^b^ | 0.35 ± 0.13^a^ | 1.48×10^-6^ |
| **%K** | 0.54 ± 0.30^a^ | 0.74 ± 0.32^a^ | 0.64 ± 0.33^a^ | 0.0754 |
| **P_2_O_5_ (mg/kg)** | 7.92 ± 7.28^a^ | 10.56 ± 5.69^a^ | 1.89 ± 1.29^b^ | 5.02×10^-7^ |

**Key: pH, acidity/basicity; EC, soil water Electrical conductivity; %TOC, percentage of total organic carbon; %O.M, percentage of organic matter; %N, percentage of available nitrogen; %K, percentage of available potassium; P_2_O_5_ (mg/kg), available phosphorus content.** **Different superscript letters indicate significant differences between treatments (p value < 0.05) according to the Tukey honest significance test (HSD).**

**Supplementary material 2: 16S rRNA sequencing statistics**

| **Study site** | **Sample ID** | **Total Reads** | **Reads Passing quality filtering** | **%Reads Passing Quality Filtering** |
| --- | --- | --- | --- | --- |
| **Gituamba** | S1 | 1,887,650 | 1,704,876 | 90.32 |
| **Mangu** | S2 | 882,248 | 786,002 | 89.09 |

**Supplementary material 3: SqueezeMeta shotgun metagenomic sequencing statistics**

| **Study site** | Ngenda |
| --- | --- |
| **Sample ID** | D3 |
| **Total Reads** | 67,136,628 |
| **Total bases** | 10,137,630,828 |
| **Mapped reads** | 27,584,941 |
| **Mapping percentage (%)** | 41.09 |
| **Number of Contigs** | 2,504,874 |
| **Total length of Contigs** | 1,097,659229 |
| **Longest Contig (bp)** | 125,240 |
| **Shortest Contig (bp)** | 128 |
| **N50** | 422 |
| **N90** | 300 |
| **Number of Open Reading Frames (ORFs)** | 3,205,501 |
| **Number of ORFs by Prodigal** | 3,019,503 |
| **KEGG annotations** | 1,508,761 |

**Supplementary material 4: Alpha diversity measures for bacterial community in Ngenda soils**

| **Diversity Metrics** | **Index Value** |
| --- | --- |
| **Shannon** | 1.765935 |
| **Simpson** | 0.487813 |

**Supplementary material 5: Relative abundance (%) of bacterial taxonomic level composition in Gituamba and Mangu soils**


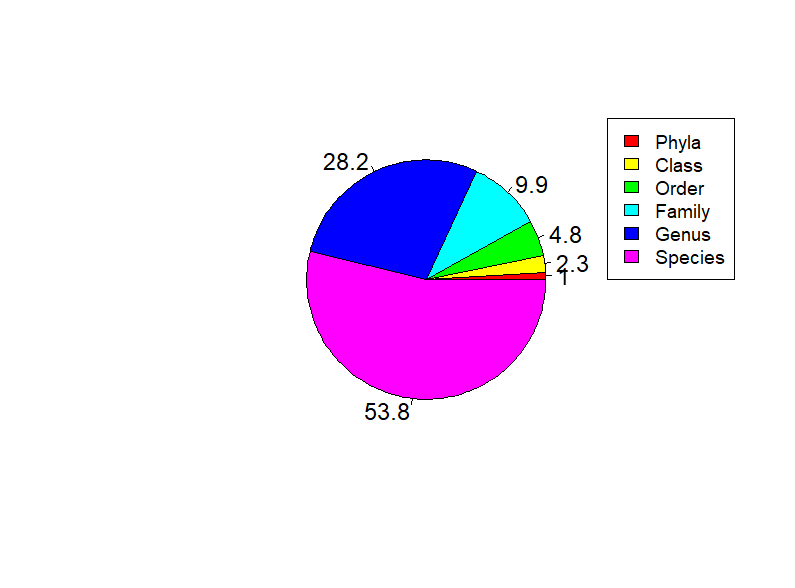


**Supplementary material 6: Relative abundance (%) of bacterial taxonomic level composition in Ngenda soils**


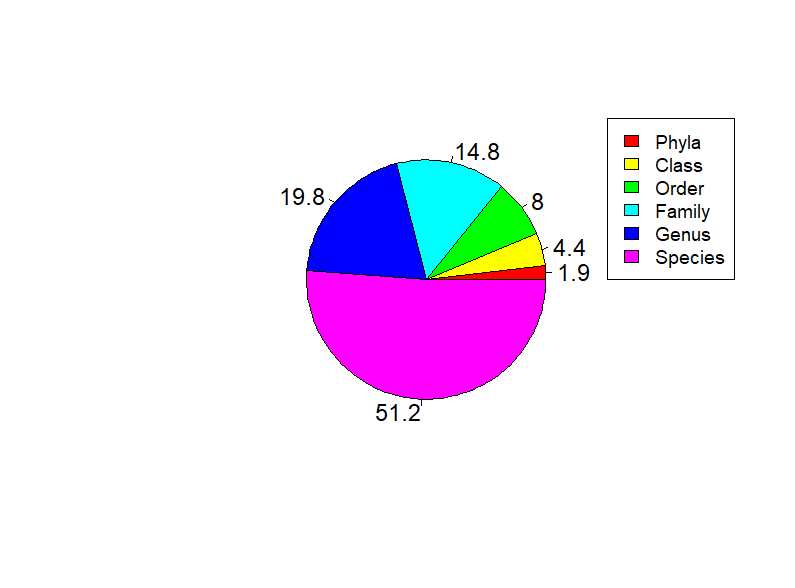

Supplement: Supplementary file 2 [file Data_Sheet_2.docx]
